# Effectiveness and Toxicity of Fractionated Proton Beam Radiotherapy for Cranial Nerve Schwannoma Unsuitable for Stereotactic Radiosurgery

**DOI:** 10.3389/fonc.2021.772831

**Published:** 2021-11-17

**Authors:** Tanja Eichkorn, Sebastian Regnery, Thomas Held, Dorothea Kronsteiner, Juliane Hörner-Rieber, Rami A. El Shafie, Klaus Herfarth, Jürgen Debus, Laila König

**Affiliations:** ^1^ Department of Radiation Oncology, Heidelberg University Hospital, Heidelberg, Germany; ^2^ National Center for Radiation Oncology (NCRO), Heidelberg Institute for Radiation Oncology (HIRO), Heidelberg, Germany; ^3^ Institute of Medical Biometry and Informatics, Heidelberg University, Heidelberg, Germany; ^4^ Clinical Cooperation Unit Radiation Oncology (E050), German Cancer Research Center (dkfz), Heidelberg, Germany; ^5^ National Center for Tumor diseases (NCT), Heidelberg, Germany; ^6^ Deutsches Konsortium für Translationale Krebsforschung (DKTK), Partner Site Heidelberg, German Cancer Research Center (dkfz), Heidelberg, Germany

**Keywords:** acoustic neuroma, vestibular schwannoma, radiation-induced contrast enhancements (RICE), pseudoprogression, radiation necrosis

## Abstract

**Purpose:**

In this benign tumor entity, preservation of cranial nerve function is of special importance. Due to its advantageous physical properties, proton beam radiotherapy (PRT) is a promising approach that spares healthy tissue. Could PRT go along with satisfactory preservation rates for cranial nerve function without compromising tumor control in patients with cranial nerve schwannoma unsuitable for stereotactic radiosurgery?

**Methods:**

We analyzed 45 patients with cranial nerve schwannomas who underwent PRT between 2012 and 2020 at our institution. Response assessment was performed by MRI according to RECIST 1.1, and toxicity was graded following CTCAE 5.0.

**Results:**

The most common schwannoma origin was the vestibulocochlear nerve with 82.2%, followed by the trigeminal nerve with 8.9% and the glossopharyngeal nerve as well as the vagal nerve, both with each 4.4%. At radiotherapy start, 58% of cranial nerve schwannomas were progressive and 95.6% were symptomatic. Patients were treated with a median total dose of 54 Gy RBE in 1.8 Gy RBE per fraction. MRI during the median follow-up period of 42 months (IQR 26–61) revealed stable disease in 93.3% of the patients and partial regression in 6.7%. There was no case of progressive disease. New or worsening cranial nerve dysfunction was found in 20.0% of all patients, but always graded as CTCAE °I-II. In seven cases (16%), radiation-induced contrast enhancements (RICE) were detected after a median time of 14 months (range 2–26 months). RICE were asymptomatic (71%) or transient symptomatic (CTCAE °II; 29%). No CTCAE °III/IV toxicities were observed. Lesions regressed during the follow-up period in three of the seven cases, and no lesion progressed during the follow-up period.

**Conclusion:**

These data demonstrate excellent effectiveness with 100% local control in a median follow-up period of 3.6 years with a promising cranial nerve functional protection rate of 80%. RICE occurred in 16% of the patients after PRT and were not or only mildly symptomatic.

## Introduction

Schwannomas are usually benign and slowly growing nerve sheet tumors that arise from the Schwann cells lining of peripheral nerves. They are most commonly located in the intradural extramedullary space and therefore affect mainly cranial or spinal nerves (1). Vestibular schwannomas (also known as acoustic neuromas) that commonly arise from the vestibular portion of the eighth cranial nerve account for most schwannomas. The overall incidence is approximately 1 in 100,000 persons per year in Western countries (2). While sporadic schwannomas are rare, they occur frequently in patients with neurofibromatosis. Bilateral vestibular schwannomas in children with neurofibromatosis put them at risk for complete deafness (3–6).

In the past decades, the most effective treatment for progressive cranial nerve schwannomas has been complete surgical resection. While local control rates were excellent, injuries of the affected or adjacent cranial nerves make the treatment of cranial nerve schwannomas a major challenge. A large surgical trial reports in vestibular schwannoma a functional hearing deterioration in up to 60.5% after surgical resection. So, cranial nerve injuries cause a relatively high morbidity compared to the excellent oncologic prognosis (7, 8). Due to the slow progression of schwannomas, the “watch and wait strategy” can be a legitimate treatment option for selected patients (9). Therapy sequelae like hearing impairment and tinnitus, vertigo and gait disturbances, facial nerve palsy, facial pain or hypesthesia, swallowing difficulties, or others also impair the quality of life. Therefore, treatment decision needs to be made carefully, and research on nerve-saving techniques is necessary.

A promising new technique was developed with stereotactic radiosurgery. Even if large prospective data are still lacking, stereotactic radiosurgery (SRS) seems to be superior regarding the risk of cranial nerve damage in schwannomas smaller than 3 cm with excellent local control rates compared to surgical resection and is therefore taken into consideration in the European Association of Neuro-Oncology (EANO) guidelines (9).

But concerns about the induction of malignant transformation of schwannomas or secondary malignancy induction after stereotactic radiosurgery still exist, even if there is no evidence for these concerns in literature, neither for stereotactic radiosurgery nor for particle beam radiotherapy (10–13). Proton beam radiotherapy (PRT) is a promising approach to better spare healthy surrounding tissue and therefore might contribute to the reduction of side effects in patients unsuitable for SRS due to the advanced tumor stage.

The major concern with proton beam radiotherapy is the risk of radiation-induced contrast enhancements (RICE), as known from, e.g., glioma trials (14, 15). RICE are defined by new brain lesions outside the tumor volume related to cerebral irradiation that are usually contrast-enhancing and not caused by the tumor. These RICE are usually transient blood-brain barrier disruptions and rarely real necrosis.

To date, there are only scarce data on PRT for schwannomas. One study investigated efficacy and toxicity rates in 94 patients who underwent fractionated PRT for vestibular schwannoma. These data demonstrated excellent local control rates and a dose-dependent risk for hearing deterioration of 36% to 56% with doses from 50.4 to 54 Gy, while the risk for damage of other cranial nerves was 5% (16). A case series supported the fact that fractionated PRT for vestibular schwannoma is well tolerated and provides good local control (17). A retrospective cohort study investigated proton (!) beam stereotactic radiosurgery and reported a 5-year tumor control rate of 95% and a dose dependency for facial neuropathy (18). No data at all exist on PRT for other schwannomas except for vestibular schwannomas.

This study investigates the effectiveness and toxicity of fractionated proton beam radiotherapy for cranial nerve schwannomas that were unsuitable for SRS. Is excellent tumor control achievable without major sequelae?

## Patients and Methods

### Patient Characteristics

**Figure 1:**
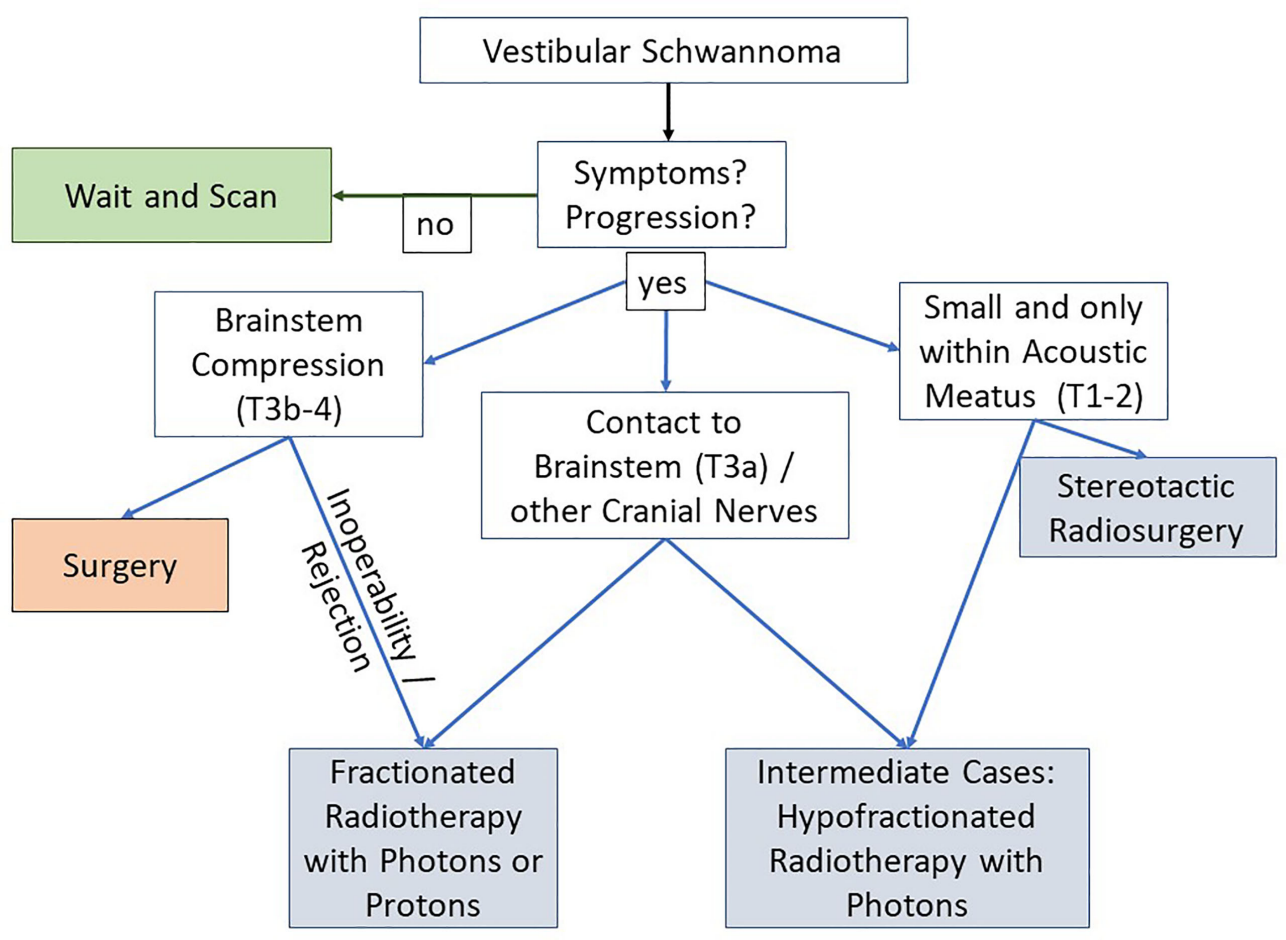
Treatment decision-making in our study cohort exemplarily for vestibular schwannoma. For other cranial nerve neuromas than vestibular schwannoma, similar decision algorithms were used.

According to its physical properties, in patients with large target volumes (for skull base schwannoma defined by T3-T4 tumors) or tumors in close proximity to the brain stem or other cranial nerves or if patients could not undergo surgery, PRT was chosen, as it is suspected that, in these cases, PRT is more suitable than stereotactic radiosurgery (SRS) and might be more suitable than fractionated photon radiotherapy. Potential physical superiority of protons over photons is well investigated in literature, but potential clinical superiority of fractionated proton radiotherapy over fractionated photon radiotherapy for cranial nerve schwannomas has never been proven. This study shall provide some clinical data on fractionated proton radiotherapy for cranial nerve schwannomas. **Figure 1** demonstrates how decision was made to treat patients with fractionated PRT. We finally included 45 patients with cranial nerve schwannomas who underwent fractionated PRT between 2012 and 2020 at our ion beam therapy center. Patient and treatment data were extracted from a clinical database maintained at our institution and from medical and official records. The first follow-up cMRI was performed 2–3 months after finishing radiotherapy. If no abnormalities were found, the following cMRIs were done in time intervals of 6–12 months thereafter. Exploration of RICE risk factors included all available treatment and patient characteristics as listed in the tables.

### Planning and Treatment Features

Immobilization was ensured by using individually shaped thermoplastic masks in the head first-supine position. In this positioning, a computed tomography (CT) scan with 3-mm slice thickness as well as a cranial magnetic resonance tomography (cMRI) with contrast were acquired for treatment planning. Gross tumor volume (GTV) comprised the contrast enhanced schwannoma in T1-weightened cMRI. A planning target volume (PTV) margin of 3 mm isotopically was added to account for geometrical uncertainties and physical beam inaccuracies. Treatment planning followed the principle of irradiation dose being as low as reasonably achievable (ALARA) without compromising PTV coverage. Dose prescription to the target volume was performed according to the constraints of ICRU report 50 and 62. Normal tissue constraints according to QUANTEC and Emami et al. (19, 20) were adhered to and sometimes adapted according to the preserved cranial nerve function, e.g., hearing function. Active beam application using raster-scanning technique with a spot size between 8 and 30 mm full width at half maximum (FWHM), with 2–3 mm of overlap in lateral (dx, dy) and longitudinal (dz) directions and synchrotron energy (48–250 MeV), was used, the active change of energy being available in 256 discrete steps, using two to three treatment beams under daily image guidance, orthogonal x-rays mounted on a ceiling robotic arm and with 2D–3D image registration for the robotic couch position correction. Either single-beam optimization (SBO) or multibeam optimization (IMPT) was used. IMPT was aimed for avoiding high-dose gradients per field, e.g., in difficult shaped targets. The final proton dose was scaled with a constant RBE factor of 1.1. Treatment was performed with five to six fractions per week. We took anatomic factors that might lead to dose uncertainties into account and used multiple beams, if needed. Furthermore, in our clinical routine, it is mandatory to regularly perform position verification scans during the treatment period with plan recalculations. So, in case of anatomical changes, e.g., in the petrous bone cavities, we adapted treatment plans. The conformity index (CI) for PTV was calculated according to the RTOG guidelines (21) by division of the CTV covered by the 95% isodose (reference isodose) and the target volume itself. A value close to 1 corresponds to ideal conformity.

### Endpoints

Trial endpoints were effectiveness and toxicity of proton beam radiotherapy. Effectiveness was evaluated by “Response Evaluation Criteria in Solid Tumors” (RECIST) version 1.1 and divided into complete or partial response, stable disease, and progressive disease (22, 23). Schwannoma progression was defined as an increase in volume according to RECIST in the follow-up period without spontaneous regression in the following cranial magnetic resonance imaging (cMRI) to distinguish it from post-treatment edema. Toxicity was graded following the National Cancers Institute’s Common Terminology Criteria for Adverse Events (CTCAE) (version 5.0). Cranial nerve functional impairment was assessed by history taking and physical exam. RICE was defined by a new post-treatment contrast enhancement in cMRI outside the GTV during the follow-up period. To face the risk of misinterpretation of RICE as tumor progression or the other way around, all images were reviewed independently by one radiologist and two radiation oncologists. Other toxic effects were assessed based on both medical records and imaging reports.

### Statistical Analysis

Descriptive statistics for baseline variables (**Tables 1**, **2**) and for objectives (**Tables 3**–**5**) include means (SD) and/or median (IQR and range, as appropriate) for continuous variables and absolute and relative frequencies for categorical variables. To identify influencing factors on clinical symptom improvement or deterioration in the follow-up period, a logistic regression model was applied using multiple patient and treatment characteristics: age > 55 years, tumor volume ≥ 5ml, technique (IMPT vs. SBO), number of beams, and fractions per week. Since this is a retrospective exploratory data analysis, p-values are of descriptive nature. Statistical analyses are performed with the software R Version 4.0.3.

**Table 1:** Patient baseline characteristics.

	n = 45 [%]	
**Gender**		
Female	23	[51.1%]
Male	22	[48.9%]
**Age at initial diagnosis (years)**		
Mean	51	
Median	51	
Standard deviation	19	
Quartile 1–quartile 3	39–66	
Minimum–maximum	10–82	
**Age at radiotherapy (years)**		
Median	55	
Minimum–maximum	18–88	
**Schwannoma risk factors**		
Neurofibromatosis type 2	5	[11.1%]
**Cranial nerve**		
Trigeminal nerve	4	[8.9%]
Vestibulocochlear nerve	37	[82.2]
Glossopharyngeal nerve	2	[4.4%]
Vagal nerve	2	[4.4%]
**Diagnostic methods**		
MRI only	28	[62.2%]
DOTATOC-PET-CT	2	[4.4%]
Partial resection/biopsy	14	[31.1%]
Complete resection	3	[6.7%]
Radiotherapy due to recurrence	3	[100%]
**Proof of progressive schwannoma before radiotherapy**		
Yes	26	[57.8%]
No	19	[42.2%]
**Symptomatic schwannoma (at radiotherapy start)**		
Yes	43	[95.6%]
No	2	[4.4%]
**Tumor size at radiotherapy start (ml)**		
Mean	8	
Median	5	
Standard deviation	10	
Quartile 1–quartile 3	3–8	
Minimum–maximum	0.3–60	

Table 2Treatment characteristics.

**Table:** 

A) Overview	n = 45 [%]	
**Primary diagnosis until radiation therapy start (months)**		
Median	18	
Minimum–maximum	2–159	
**Total dose (Gy RBE)**		
Mean	54	
Median	54	
Standard deviation	3	
Quartile 1–quartile 3	54–56	
Minimum–maximum	40–58	
**dose per fraction (Gy RBE)**		
Mean	1.8	
Median	1.8	
Standard deviation	0.2	
Quartile 1–quartile 3	1.8–1.8	
Minimum - maximum	1.8–2	
**Number of fractions**		
Mean	29	
Median	30	
Standard deviation	3	
Quartile 1–quartile 3	30–31	
Minimum–maximum	15–32	
**Fractions per week**		
5 fractions per week	23	[51.1%]
6 fractions per week	22	[48.9%]
**Proton beam radiotherapy technique**		
IMPT	37	[82.2%]
SBO	8	[17.8%]
**Number of beams**		
1	2	[4.4%]
2	27	[55.6%]
3	19	[40.0%]
**GTV (ml)**		
Mean	8	
Median	5	
Standard deviation	10	
Quartile 1–quartile 3	3–8	
Minimum–maximum	0.3–60	
**PTV (ml)**		
Mean	20	
Median	12	
Standard deviation	22	
Quartile 1–quartile 3	20–23	
Minimum–maximum	4–137	

Dmax, maximum dose; Dmean, mean dose; Gy RBE, gray relative biological effectiveness.

**Table:** 

B) Doses for organs at risk (Gy RBE)	Dmax	Dmean
**Inner ear**		
Mean	49.6	40.4
Median	54	43.8
Quartile 1–quartile 3	51.1–55.4	38–50.3
Minimum–maximum	0–59.4	0–57.3
**Adjacent cranial nerves**		
Mean	8.6	3.1
Median	2.4	0.4
Quartile 1–quartile 3	0.9–9.3	0.1–1.2
Minimum–maximum	0.3–51.9	0–45.5
**Ipsilateral temporal lobe**		
Mean	50.3	13.6
Median	53.2	13.6
Quartile 1–quartile 3	50.3–54.8	12.2–16.4
Minimum–maximum	3.7–58.3	0–19.8
**Brain stem**		
Mean	51.4	12
Median	53.5	10.6
Quartile 1–quartile 3	52–54.6	6.1–14.9
Minimum–maximum	20.3–57.9	0.5–44.9
**Ventricular system**		
Mean	55.1	6.1
Median	55.1	6.1
Quartile 1–quartile 3	55.1–55.1	6.1–6.1
Minimum–maximum	55.1–55.1	6.1–6.1

Dmax, maximum dose; Dmean, mean dose; Gy RBE, Gray Relative Biological Effectiveness.

**Table 3:** Treatment effectiveness observation.

	n = 45 [%]	
**Time PRT end until first imaging follow-up (weeks)**		
Mean	9	
Median	8	
Standard deviation	9	
Quartile 1–quartile 3	6–10	
Minimum–maximum	1–44	
**Total follow-up period (months)**		
Mean	43	
Median	42	
Standard deviation	25	
Quartile 1–quartile 3	26–61	
Minimum–maximum	3–97	
**Response to radiotherapy during follow-up period**		
Complete remission	0	[0.0%]
Partial remission	3	[6.7%]
Stable disease	42	[93.3%]
Progressive disease	0	[0.0%]
**Progression-free survival**		
Yes	45	[100%]
No	0	[0.0%]

**Table 4:** Long-term treatment toxicity assessment.

Clinical symptom	Pre-irradiation				Early post-irradiation				Late post-irradiation				Overall post-irradiation			
	Low grade* (CTCAE I-II)		High grade* (CTCAE ≥III)		Low grade (CTCAE I-II)		High grade (CTCAE ≥III)		Low grade (CTCAE I-II)		High grade (CTCAE ≥III)		Improvement		deterioration	
	n	[%]	n	[%]	n	[%]	n	[%]	n	[%]	n	[%]	n	[%]	n	[%]
Any	43	[95.6%]	0	[0.0%]	45	[100%]	0	[0.0%]	45	[100%]	0	[0.0%]	0	[0.0%]	16	[35.6%]
Cranial nerves																
Olfactory nerve	0	[0.0%]	0	[0.0%]	0	[0.0%]	0	[0.0%]	0	[0.0%]	0	[0.0%]	0	[0.0%]	0	[0.0%]
Optic nerve	0	[0.0%]	0	[0.0%]	0	[0.0%]	0	[0.0%]	0	[0.0%]	0	[0.0%]	0	[0.0%]	0	[0.0%]
Oculomotory nerve	0	[0.0%]	0	[0.0%]	0	[0.0%]	0	[0.0%]	0	[0.0%]	0	[0.0%]	0	[0.0%]	0	[0.0%]
Trochlear nerve	0	[0.0%]	0	[0.0%]	0	[0.0%]	0	[0.0%]	0	[0.0%]	0	[0.0%]	0	[0.0%]	0	[0.0%]
Trigeminal nerve	7	[15.6%]	0	[0.0%]	9	[20.0%]	0	[0.0%]	9	[20.0%]	0	[0.0%]	0	[0.0%]	3	[6.7%]
Abducens nerve	0	[0.0%]	0	[0.0%]	0	[0.0%]	0	[0.0%]	0	[0.0%]	0	[0.0%]	0	[0.0%]	0	[0.0%]
Facial nerve	11	[26.7%]	0	[0.0%]	12	[26.7%]	0	[0.0%]	12	[26.7%]	0	[0.0%]	0	[0.0%]	1	[2.2%]
Vestibulocochlear nerve	37	[82.2%]	0	[0.0%]	37	[82.2%]	0	[0.0%]	37	[82.2%]	0	[0.0%]	2	[4.4%]	5	[11.1%]
Tinnitus	11	[22.4%]	0	[0.0%]	12	[26.7%]	0	[0.0%]	12	[26.7%]	0	[0.0%]	1	[2.2%]	5	[11.1%]
Hearing impairment	36	[80.0%]	0	[0.0%]	36	[80.0%]	0	[0.0%]	36	[80.0%]	0	[0.0%]	0	[0.0%]	0	[0.0%]
Vertigo	16	[35.6%]	0	[0.0%]	16	[35.6%]	0	[0.0%]	16	[35.6%]	0	[0.0%]	2	[4.4%]	3	[6.7%]
Glossopharyngeal nerve	1	[2.2%]	0	[0.0%]	1	[2.2%]	0	[0.0%]	1	[2.2%]	0	[0.0%]	0	[0.0%]	0	[0.0%]
Vagal nerve	1	[2.2%]	0	[0.0%]	1	[2.2%]	0	[0.0%]	1	[2.2%]	0	[0.0%]	0	[0.0%]	0	[0.0%]
Accessory nerve	0	[0.0%]	0	[0.0%]	0	[0.0%]	0	[0.0%]	0	[0.0%]	0	[0.0%]	0	[0.0%]	0	[0.0%]
Hypoglossal nerve	0	[0.0%]	0	[0.0%]	0	[0.0%]	0	[0.0%]	0	[0.0%]	0	[0.0%]	0	[0.0%]	0	[0.0%]
RICE	0	[0.0%]	0	[0.0%]	0	[0.0%]	0	[0.0%]	7	[15.6%]	0	[0.0%]	0	[0.0%]	7	[15.6%]
Others																
Fatigue	0	[0.0%]	0	[0.0%]	11	[24.4%]	0	[0.0%]	3	[6.7%]	0	[0.0%]	0	[0.0%]	11	[24.4%]
Headache	0	[0.0%]	0	[0.0%]	5	[11.1%]	0	[0.0%]	1	[2.2%]	0	[0.0%]	0	[0.0%]	5	[11.1%]
Skin toxicity	0	[0.0%]	0	[0.0%]	1	[2.2%]	0	[0.0%]	0	[0.0%]	0	[0.0%]	0	[0.0%]	1	[2.2%]
Dysgeusia	0	[0.0%]	0	[0.0%]	1	[2.2%]	0	[0.0%]	0	[0.0%]	0	[0.0%]	0	[0.0%]	1	[2.2%]
Alopecia	0	[0.0%]	0	[0.0%]	4	[8.9%]	0	[0.0%]	0	[0.0%]	0	[0.0%]	0	[0.0%]	4	[8.9%]

Assessment based on medical and physical exam. Early post-irradiation symptoms were assessed in median after 8 (Q1–Q3: 6–10) weeks, and late post-irradiation symptoms were assessed during the total follow-up period (median, Q1–Q3: 42, 26–31 months). RICE, radiation-induced contrast enhancements. *All documented signs and symptoms were graded analogous to CTCAE grading for better comparability. CTCAE, common terminology criteria for adverse events.

Table 5Detailed analysis of RICE (n=7 [15.6%]).

**Table:** 

A) Overall RICE analysis		
	n = 7 [16%]	
**Time radiotherapy end to first occurrence of RICE (months)**		
Mean	14	
Median	14	
Quartile 1–quartile 3	12–16	
Minimum–maximum	2–26	
**Symptomatic RICE**		
Yes	2 (CTCAE °II)	[28.6%]
No	5	[71.4%]
**Treatment needed for RICE**		
Steroids only	7	[100%]
Bevacizumab (anti-VEGF antibody)	0	[0%]
**Observed regression during follow-up period**		
Yes	3	[42.9%]
No	4	[57.1%]
**Observed progression during follow-up period**		
Yes	0	[0%]
No	7	[100%]

RICE, radiation-induced contrast enhancements.

**Table:** 

B) Analysis of specific RICE cases		
Case number	1	2	3	4	5	6	7
Gender	Female	Male	Female	Male	Female	Female	Female
Age at radiotherapy (years)	63.9	82.4	67.7	62.8	79.3	77.8	44.3
Latency (months)	17	14.9	2.3	13.8	25.7	10.3	13.3
Treatment	Steroids	Steroids	Steroids	Steroids	Steroids	Steroids	Steroids
Inpatient treatment needed	No	No	No	No	No	No	No
Cranial nerve	Vestibulocochlear nerve	Vestibulocochlear nerve	Vestibulocochlear nerve	Vestibulocochlear nerve	Vestibulocochlear nerve	Vestibulocochlear nerve	Vestibulocochlear nerve
Previous surgery	No	No	No	No	No	No	Yes
Neurofibromatosis type II	No	No	No	No	No	No	No
Any pre-irradiation symptoms	Yes	Yes	Yes	Yes	Yes	Yes	Yes
Pre-irradiation tinnitus	No	No	No	No	No	Yes	Yes
Pre-irradiation hearing impairment	Yes	Yes	Yes	Yes	Yes	Yes	Yes
Pre-irradiation vertigo	No	Yes	No	No	No	Yes	Yes
Any post-irradiation deterioration	No	Yes	No	No	No	Yes	No
Total dose (Gy RBE)	54.0	54.0	54.0	57.6	57.6	54.0	57.6
Dose per fraction (Gy RBE)	1.8	1.8	2.0	1.8	1.8	1.8	1.8
Fractions per week	5	5	6	5	6	6	5
Radiotherapy technique	IMPT	SBO	IMPT	IMPT	IMPT	IMPT	IMPT
Schwannoma size/GTV (ml)	5	5	7	4	5	4	2
Schwannoma maximal diameter (mm)	22	25	26	22	21	24	26
PTV (ml)	10	12	20	9	12	9	5
Maximum dose GTV (Gy RBE)	55.5	55.5	56.1	60.8	56.4	55.9	59.3
Maximum dose brain stem (Gy RBE)	53.2	53.3	54.3	54.7	52.8	52.9	55.5
Number of beams	3	2	2	3	3	3	3
Any pre-irradiation in the past	No	No	No	No	No	No	No

RICE, radiation-induced contrast enhancements.

**Table:** 

C) Logistic regression analysis for RICE risk factors		
Variable	Estimate (95% CI)	p-value
Age >55 years	-2.41 (5.77 - -0.42)	0.68
Tumor volume ≥5ml	-1.84 (1.24 - 1.48)	0.14
Technique (IMPT vs. SBO)	-0.61 (1.50 - -0.40)	0.69
Number of beams	1.77 (1.17 - 1.51)	0.13
Fractions per week	-0.72 (1.01 - -0.72)	0.47

CI, confidence interval IMPT, intensity-modulated proton therapy; SBO, single beam optimization; RICE, radiation-induced contrast enhancements.

## Results

### Patient and Treatment Characteristics

Median patient age at the beginning of radiotherapy (RT) was 55 years (range: 18–88). Gender was equally distributed. The most common schwannoma origin was the vestibulocochlear nerve with 82.2%, followed by the trigeminal nerve with 8.9% and the glossopharyngeal nerve as well as the vagal nerve, each with 4.4%. A neurofibromatosis type 2 as a risk factor for schwannoma occurrence was found in 11.1% of the patients. Between primary diagnosis and radiotherapy start passed in a median of 18 months (range: 2 months to 12 years). Diagnosis was made mainly by imaging with MRI. In 37.8% of the cases, histology was confirmed by a prior biopsy or resection. In two cases (4.4%), a DOTATOC-PET-CT was needed to exclude meningioma. In 57.8% of the patients, treatment was indicated due to observed progression. About 95.6% of all treated patients complained restrictions in everyday life due to schwannoma symptoms. In median, schwannoma size or gross target volume (GTV) was 5 ml at radiotherapy start ranging from 0.3 to 60 ml. The planning target volume (PTV) was 12 ml in median, ranging from 4 to 137 ml. The median total dose was 54 Gy (range: 40–57.6 Gy) with a median single dose of 1.8 Gy (range 1.8–2 Gy), applied in five to six fractions per week. Two patients were treated with reduced doses due to pre-irradiation in the past. The most commonly used technique was intensity modulated proton therapy (IMPT) with 82.2%, followed by single beam optimization (SBO) with 17.8%. Between one and three beams were used with two beams in 60.0%, three beams in 42.2%, and one beam in 4.4%.

Detailed patient characteristics are presented in **Table 1**; detailed treatment characteristics are presented in **Table 2**.

### Efficacy and Toxicity

The follow-up period was 42 months in median (IQR 26–61). In median after 2 months (range: 0.25–11 months), the first follow-up MRI was performed. During the entire follow-up period, 93.3% of the patients showed stable disease, and 6.7% demonstrated partial remission. None of the patients had a schwannoma progression in the observation period. Detailed data on treatment effectiveness observation are presented in **Table 3**.

Before radiotherapy start and during the follow-up period, detailed data on clinical signs and symptoms caused by schwannoma were recorded *via* repeated medical history and physical exam assessment at each follow-up visit. This allows for a longitudinal presentation in the course of time of multiple specific symptoms as presented in **Table 4**. At radiotherapy start, 95.6% reported clinical symptoms due to the schwannoma. With vestibular schwannoma being predominantly observed, most patients complained about hearing impairment (80.0%), followed by vertigo (35.6%) and tinnitus (22.4%). A trigeminal neuralgia was reported by 15.6% of the patients, and 4.4% of the patients suffered from difficulty swallowing. All symptoms were graded analogous to CTCAE grading for better comparability with follow-up findings even if CTCAE grading was not developed to assess pretherapeutic symptoms. After PRT, 60% of the patients had stable symptoms, 4.4% of the patients reported a symptom improvement, and 35.6% of the patients reported any symptom deterioration after PRT, mainly transient fatigue. New or worsening cranial nerve dysfunctions were found in 20.0% of all the patients in the follow-up period, e.g., a worsening in tinnitus, but never relevant for activities of daily life. The reported symptoms described above were mild and graded CTCAE I-II. No CTCAE °III/IV toxicities were observed. Symptoms and toxicities that were not associated to cranial nerves like fatigue, headache, skin toxicity, dysgeusia, and alopecia were mainly observed in the early post-irradiation period and resolved during further follow-up. Explorative analysis *via* logistic regression modeling could not find any substantially influencing factors among all descriptively presented variables on the improvement or deterioration of clinical symptoms.

No secondary malignancies were encountered during the follow-up period.

### RICE

The follow-up MRIs revealed a contrast enhancement in brain parenchyma after PRT consistent with an RICE in seven cases (15.6%). These lesions were observed after a median of 14 months (range: 2–26 months) after PRT. In two of the seven RICE cases (29%), a deterioration of clinical symptoms was observed; in one case, mild gait disturbances and in the other case a facial paresthesia were reported, which made an outpatinet treatment with a short course of orally administered necessary (both CTCAE °II). Due to sufficient response to corticosteroid treatment, in no case bevacizumab administration as an anti-VEGF antibody was needed. Three of the seven lesions (43%) regressed during the follow-up period, and no lesion showed progression. Further analysis of RICE cases did not reveal considerable differences in the subgroup, in which RICE occurred, compared to the subgroup, in which no RICE was diagnosed. Patients with RICE had been treated with standard therapy of 54–57.6 Gy in 1.8–2.0 Gy RBE per fraction with five to six fractions per week. GTV volume was 2–7 ml. Only one patient underwent previous surgery; none of the patients had neurofibromatosis type 2. CI for PTV was in the median of 0.99 (range 0.98–1.0). Due to lack of power, explorative analysis *via* logistic regression modeling could not find any influencing factors on the development of RICE by comparing the 7 RICE cases to the 38 cases without RICE among the tested variables: age > 55 years, tumor volume ≥5 ml, technique (IMPT vs. SBO), number of beams, and fractions per week. **Table 5** presents a detailed workup of RICE. **Table 5A** demonstrates an overview analysis on all RICE cases, whereas **Table 5B** shows a detailed descriptive analysis of each specific RICE case. **Table 5C** shows the logistic regression model for RICE risk factors. **Figure 1** shows representative images for all seven cases.

## Discussion

This study investigates the effectiveness and toxicity of fractionated proton beam radiotherapy for cranial nerve schwannomas.

As expected, most cranial nerve schwannomas affected the vestibulocochlear nerve, and therefore, most patients presenting to our clinic suffered from hearing impairment, tinnitus, or vertigo. Nevertheless, cranial nerve schwannomas originating from the trigeminal nerve, the glossopharyngeal nerve, and the vagal nerve were also found, mainly associated to neurofibromatosis type 2 as supported by literature data (24). Most patients sought for treatment due to clinical symptoms or a documented schwannoma progression under monitoring.

Proton beam radiotherapy for cranial nerve schwannoma was shown to be effective. No schwannoma progression was observed, and therefore, 100% effectivity can be reported for the follow-up period of 3.5 years in median. These effectivity rates for proton beam radiotherapy are very promising. However, further prolongation of the follow-up period is needed. The only data available on PRT in vestibular schwannoma include 95 patients treated from March 1991 to March 2008 at Loma Linda University Medical Center. Fractionated proton radiotherapy at daily doses of 1.8 Gy and a total dose ranging between 59.6 Gy (RBE) and 50.4 Gy (RBE) was employed, depending on hearing function. Local control rates for the median follow-up time of 64 months were 95–92%, depending on the applied dose. Cranial nerve injuries occurred in two patients. Hearing preservation was maintained in 44–64% of the patients, depending on the applied dose. The overall patient cohort was divided into three groups with graduated dose concepts (16).

For gamma knife, a control rate of 98% after a median follow-up period of 5.8 years was reported (25). For the “watch-and-wait” strategy, a meta-analysis demonstrated in median 43% of schwannoma progressions in a similar follow-up period of 3.2 years (26). For surgery, a median of 9% schwannoma progression was demonstrated in a follow-up of 3.1 years (27) with a considerable rate of surgery-associated cranial nerve damage with chances of hearing preservation between 47% and 88% and even death due to surgery-associated complications (28). Therefore, as stated by the EANO Guideline on the Diagnosis and Treatment of Vestibular Schwannoma, radiotherapy is superior to both the “watch-and-wait” strategy regarding efficacy as well as surgery especially regarding toxicity in small tumors (<3 cm) (9). But both the “watch-and-wait” strategy and surgery are valuable for selected patients. Patients without any symptoms or progression may be suitable for the “watch-and-wait” strategy, while very large tumors that have already completely destroyed the nerve and compress the brainstem may be suitable for surgery. For radiotherapy, there are several factors, e.g., dose, fractionation regimes, and treatment techniques, that have to be taken into consideration when deciding about the individually best approach. According to a retrospective comparative analysis of 125 patients in total, hearing preservation rates might be 2.5-fold higher in fractionated radiotherapy than in single fraction stereotactic radiosurgery, but tumor control rates were at least 97% in both groups (29). However, literature demonstrates also data that report similar rates for hearing preservation and local control for both fractionated radiotherapy and single fraction stereotactic radiosurgery, and the dose of single fraction stereotactic radiosurgery was significantly influencing hearing preservation (30). Furthermore, according to literature, it is recommended to use single fraction stereotactic radiosurgery for smaller lesions, while FSRT can be used independently of tumor size and should be preferred in larger tumors (with contact to the brainstem) due to the better adherence to dose constraints with FSRT (30–33). For PRT, fractionated radiotherapy might be less toxic than single fraction stereotactic radiosurgery (16, 18). We cannot directly compare our results to stereotactic radiosurgery as our cohort was defined by unsuitability for stereotactic radiosurgery (T3-T4 tumors). Valid treatment alternatives for our cohort were surgery or fractionated photon radiotherapy. Cranial nerve preservation of the affected cranial nerve in fractionated proton/photon radiotherapy is well known to be superior to surgery. Compared to fractionated photon radiotherapy, fractionated proton radiotherapy spares the adjacent cranial nerves due to steep dose gradients given in proton radiotherapy. This is a dosimetric advantage, and literature is lacking data whether this results in clinically detectable advantages. Since cranial nerve neuromas are mainly located at the skull base often in close proximity to the brain stem or the facial nerve (for acoustic neuroma/vestibular schwannoma), sparing of adjacent structures is critical.

Interestingly, a retrospective analysis found lower tumor control rates in patients with neurofibromatosis type 2 compared to sporadic tumors (29). This was not confirmed by our study using fractionated PRT.

When patients present for radiotherapy, they are usually suffering from schwannoma-associated symptoms that are mainly hearing impairments in vestibular schwannoma. None of our patients reported a new or worsening hearing impairment after PRT despite relevant dose deposition on the inner ear (D_mean_ in median = 43.8 Gy). Nevertheless, data on hearing function are based on medical history and physical exam so a very mild hearing deterioration might be underestimated in this analysis, since audiometry was not performed on a regular basis but only if symptoms were reported. Nevertheless, the used method is appropriate to detect a hearing deterioration that is in any way relevant to the patients’ daily life. One-fifth of the patients reported any new or worsening mild clinical symptoms associated to the affected cranial nerve in the follow-up period with a worsening tinnitus being the most common observation. Symptoms like tinnitus or vertigo might represent an irritation of the irradiated cranial nerve. Even if some patients (4.4%) reported an improvement of clinical symptoms, this analysis demonstrates that PRT can irritate or slightly impair the function of the affected cranial nerve leading to a mild deterioration of symptoms even if no loss of cranial nerve function was observed.

To sum up the scarce available data, fractionated proton beam radiotherapy for tumors unsuitable for stereotactic radiosurgery is a promising approach that should be further investigated. For detailed assessment of hearing function using repetitive audiometry, a prospective clinical trial is urgently needed. Such a trial is currently conducted at Boston using fractionated proton radiation therapy for vestibular schwannomas and will close recruitment in a couple of months, so results will be pending for multiple years (ClinicalTrials.gov Identifier: NCT01199978).

**Figure 2:**
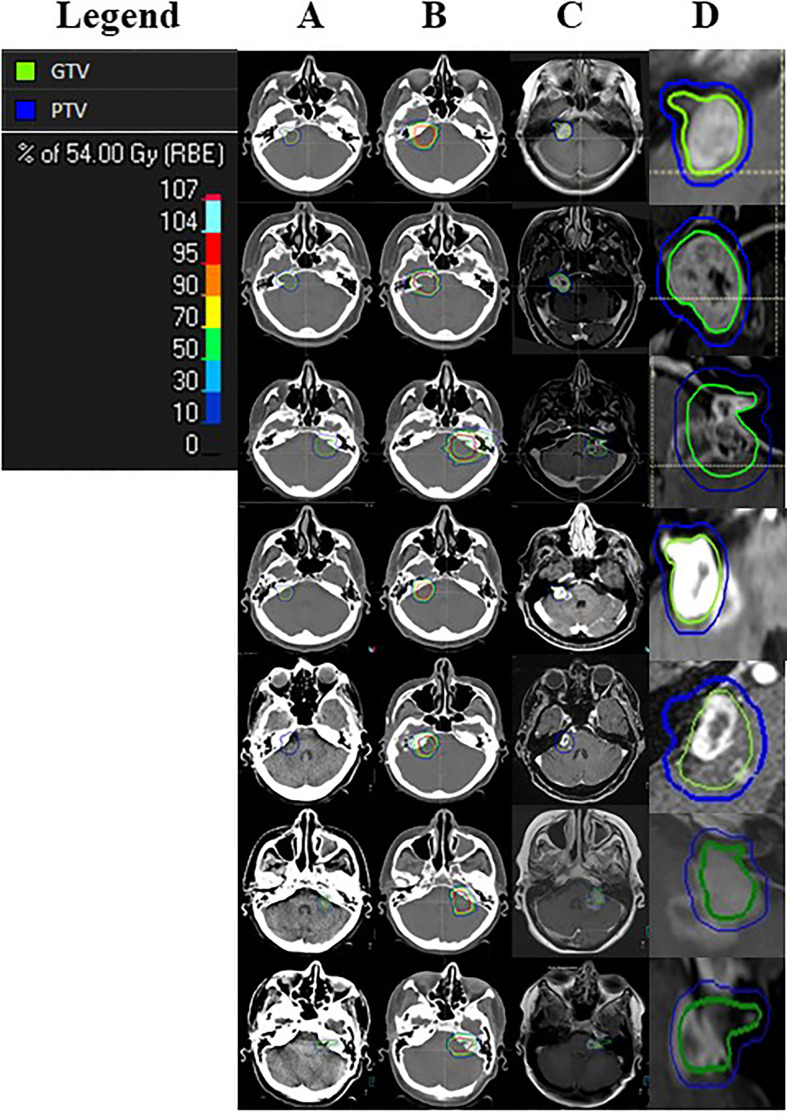
Cases of Radiation-induced Contrast Enhancements (RICE). The image presents all seven cases of RICE observed in our study cohort. Column A presents the planning computer tomography with gross target volume (GTV, green) and planning target volume (PTV, blue) for each patient. Column B demonstrates the treatment plan with isodoses for each patient. Column C presents the follow-up magnetic resonance imaging (MRI) with the observed RICE at the time of its first notice and a projection of the initial GTV and PTV in this MRI with an enlargement of this region (column D).

A special feature of this study is that RICE has been defined as an endpoint, and it was specifically evaluated for its occurrence, clinical presentation, treatment, and time course. Despite all advantageous properties of proton beam radiotherapy presented above, the rate of 16% for RICE needs to be further focused on. Due to close proximity of the irradiated cranial nerve schwannomas to the brain stem, RICE occurred in the vulnerable healthy brain stem tissue (**Figure 2**). This unfavorable localization was also the reason why RICE treatment was administered liberally, so all of our RICE cases received a short course of corticosteroids. In two (of seven) patients, RICE were mild symptomatic, but corticosteroids relieved symptoms quickly, so none of the patients needed a bevacizumab therapy. Anti-VEGF antibodies like bevacizumab go along with excellent remission rates for RICE and are therefore the treatment of choice in more severe cases of RICE (34, 35). We showed that these lesions occurred after a median of 14 months. In 43%, the lesions regressed during the follow-up period. Due to this observed time course, we recommend a close timeline of follow-up including MRI within the first 2 years. For these 43% of cases, RICE is probably representing a radiation-induced blood-brain barrier disruption and probably not an irreversible radiation necrosis due to its transient nature, as supported by literature (36).

While the applied dose is standard (16), nearly half of our patients received up to six fractions per week, which is due to historical reasons and can therefore be regarded as slight acceleration. We decided at our institution not to proceed the irradiation with six fractions per week for central nervous system tumors as a safety measure. Nevertheless, this was not an influencing factor in the performed exploratory analysis regarding toxicity, RICE, or response of the treatment, but power was lacking. PTV conformity was sufficient in these cases, so inconformity can be excluded as a potential cause for RICE.

Another hypothesis is that the current technique of dose calculation for proton beam radiotherapy underestimates the real regional biological effectiveness as currently heavily discussed in the proton beam radiotherapy community. Interestingly, an RICE localization exactly behind the peak and therefore exactly distally to the target volume is a typical finding (as also depicted in **Figure 1**). The current method of dose prescription in PRT has known weaknesses. This leads to an uncertainty in dose calculation and therefore to underdosed and overdosed areas. Possibly, also an intrinsic difference in radiosensitivity of different brain tissue types might play a role and makes adaptions in proton beam radiotherapy planning necessary. First approaches for a physical risk model to estimate the probability of the occurrence of RICE for low-grade gliomas can be found in the literature (15). To reduce the risk for RICE without compromising the excellent oncologic outcomes, PRT planning needs to be improved further.

In summary, PRT was demonstrated to be effective and yielded high rates of cranial nerve functional preservation. The comparably low cranial nerve toxicity rates are promising, but the follow-up period needs to be further expanded. Also, a longer follow-up is needed. The analysis of RICE in this context is described for the first time. Even if no clinical relevance of RICE occurrence was demonstrated and corticosteroid response was good, it is above our understanding why some patients develop RICE and therefore further research is needed. Patients should undergo MRIs on a regular basis after cranial PRT.

## Conclusion

These data demonstrate excellent effectiveness with 100% local control in a median follow-up period of 3.6 years with a promising cranial nerve functional protection rate of 80%. RICE occurred in 16% of the patients after PRT and were not or only mildly symptomatic but made a short course of dexamethasone necessary.

## Data Availability Statement

The raw data supporting the conclusions of this article will be made available by the authors, without undue reservation.

## Ethics Statement

The studies involving human participants were reviewed and approved by Heidelberg University ethics committee, January 2018 (#S-832/2018). Written informed consent for participation was not required for this study in accordance with the national legislation and the institutional requirements.

## Author Contributions

TE, LK, KH, and JD planned and supervised this analysis. TE performed data extraction and review. TE, RES, and DK performed all statistical analysis. TE reviewed data analysis and drafted the manuscript. All authors contributed patient data and participated in reviewing and improving analysis and manuscript. All authors read and approved the final manuscript.

## Funding

We acknowledge financial support by Deutsche Forschungsgemeinschaft within the funding program for Open Access Publishing, by the Baden-Württemberg Ministry of Science, Research and the Arts and by Ruprecht-Karls-Universität Heidelberg. TE received funding by Ruprecht-Karls Universität Heidelberg, Herbert Kienzle Foundation, and Else Kröner-Fresenius Foundation.

## Conflict of Interest

TE reports grants from Ruprecht-Karls Universität Heidelberg, Herbert Kienzle Foundation, and Else Kröner-Fresenius Foundation and received travel reimbursement from Bristol-Myers Squibb outside the submitted work. JH-R received speaker fees and travel reimbursement from ViewRay Inc, as well as travel reimbursement and grants from IntraOP Medical and Elekta Instrument AB outside the submitted work. RS reports grants from Ruprecht-Karls Universität Heidelberg, during the conduct of the study; personal fees from Accuray Inc., personal fees from AstraZeneca GmbH, personal fees from Bristol Myers Squibb GmbH & Co., personal fees from Novocure GmbH, personal fees from Merck KGaA, personal fees from Takeda GmbH, and grants from Accuray Inc., outside the submitted work. JD reports grants from the Clinical Research Institute (CRI), grants from View Ray Incl., grants from Accuray International, grants from Accuray Incorporated, grants from RaySearch Laboratories AB, grants from Vision RT limited, grants from Merck Serono GmbH, grants from Astellas Pharma GmbH, grants from Astra Zeneca GmbH, grants from Siemens Healthcare GmbH, grants from Solution Akademie GmbH, grants from Eromed PLC Surrey Research Park, grants from Quintiles GmbH, grants from Pharmaceutical Research Associates GmbH, grants from Boehringer Ingelheim Pharma GmbH Co, grants from PTW-Frieburg Dr. Pychlau GmbH, grants from Nanobiotix A.a., grants from IntraOP Medical, outside the submitted work. LK reports grants from Ruprecht-Karls Universität Heidelberg, personal fees from Accuray Inc., and Novocure GmbH outside the submitted work.

The remaining authors declare that the research was conducted in the absence of any commercial or financial relationships that could be construed as a potential conflict of interest.

## Publisher’s Note

All claims expressed in this article are solely those of the authors and do not necessarily represent those of their affiliated organizations, or those of the publisher, the editors and the reviewers. Any product that may be evaluated in this article, or claim that may be made by its manufacturer, is not guaranteed or endorsed by the publisher.
